# Half-yearly Abstract of the Medical Sciences

**Published:** 1851-05

**Authors:** 


					﻿Авт. XIII.—The Half-yearly Abstract of the Medical Sciences: being
a practical and analytical digest of the contents of the principal Bri-
Łish and continental medical works published during the preceding
six months. Together with a series of critical reports on the progress
of medicine and the collateral sciences during the same period. Ed-
ited by W. H. Ranking, Μ. D., Cantab., assisted by W. A. Guy,
Μ. D., George Day, Μ. D., Henry Ancell, Μ. D., and W.
Kirkes, Μ. D. No. 12—July to December, 1850. Philadelphia:
Lindsay and Blakiston.
Of the excellent character of this publication it is not
necessary for us to say much. It has sustained itself in
England for six years, and is worthy of the patronage it
has received. It gives a very fair and useful record of
the important movements of medical science, and it should
be the companion of all medical practitioners. We com-
mend the work to the favor of the reader.	B.
Annual Report of the Louisville Marine Hospital, for
the year ending March 1st, 1851.—We have received a
copy of this report, and present our readers with the
names of the medical officers of the Institution, and a
copy of Table No. 5, containing a medical history of the
establishment, for the past year.
Physicians on part of Medical Schools.—Professors Paul
F. Eve and Samuel Annan.
Consulting Physicians.—T. S. Bell, Μ. Ð., C. Pirtle,
Μ. D., and J. W. Bright, Μ. D.
Consulting Surgeons.—T. W. Colescott, Μ. D., Ł. Pow-
ell, Μ. Ð., and S. B. Richardson, Μ. D.
Attending Surgeons.—B. I. Raphael, Μ. I)., J. Μ. Pyles,
Μ. D., and T. G. Richardson, Μ. D.
Attending Physicians.—D. W. Yandell, Μ. D., C. L.
Lyle, Μ. D., and W. B. Caldwell, Μ. D.
Resident Graduates.—Jonathan Clark, M.D., and James
.Selby, Μ. Ð.
REPORT of the Diseases and their Results, coincident with the Admissions and
Discharges at the Louisville Marine Hospital, from March 1st, 1850, to March
\st, 1851.
ADMISSIONS.	DISCHARGES.
řo £ ¦>|g	£	¦ŗ■' >	œθ|2tJ¦į<¡4ļ	d	О	מ¡ fð' И	ö ►ĵ	fβ
в 3P	ŝ	¶i	's s I § й	I	г	Hi	S-£	в
"S Γ .	·	■	■> й	в a В s Ę §	>	ř	5 S p·	: M	04
“ s H Ĳ i ! ΓFJ?/? î ·nN : | ־
Ξ ·	·	·	·	’·	'	î t î í «’ ¡ ·	S’	: . í ·	·	rjq	Į-ж
-	*	·	·	·	!	 ............ מ>	י ·.י! ם	-
g ·	¦	;	;	'·	*	····■י	·º	·	*	· î	;	·º	ļ“’1
Abscess.........  1	2 1	1	1	3	1	10	51	1	2	91
Albumenuria...... 1	1	11
Amaurosis........ 1	1	2	11	2
Amenorrhea...... •1	1	1	1
Astθienia^ ״״ľ.ľ.ľ.	7 2 1 2 2 t 248144	33 25	12	28 5
Ascites......... Ill	32	13
Bronchitis....... 3	2 1	1	1	2	1 3 4	18	11 2	1	1	15 3
Catarrh......... 3	33	3
Carcinoma....... ,2	1	4	42	1	1	4
Cardiac disease. lit	311	13
Calculus........ 1	4 I	1	·>
Caries.......... 2	111	52	132
Cholera Asiatic.	1 5 11 2(5 12	1 3 6 3	68 20	48	68į
Colica.......... 2	1	3	3	3
Colica Pictoneum..	I	11	¿ İ	,
Convulsions............................... 1
Cutanei......... 1	2 2	1	Ŝ 1	9	5 2	7 2
Constipation.... 2	1	3 đ	3
Cystitis..-...... I	2	4	1	!	5	5
^ãxfΞΞ 8■ י и ■t a ?5 5 4J s ь : ־ s1
Epilepľy11 2 1 4 1 2 2 1 1 1 :״״.■"-״ J	12	2 í ' /31
Encephalitis................. 1211	2	7	4	1161
ErvĴipells“1....	2	1	1	3	1 2	1	2 4	13	10	1	11	2
r2״ξ ............ 5	12	12	8	5	12	23	15 7	7	6	15 13	140	109	1	1	2	2j	134	e
Feve” intermittent/	14 4 7	7	11	27	29	27 10	9	11	5 5	166	155	3	2	1	161	5
ISr¿¿ľ1 17 2 1 14 18 1 1 2 ¦4 1 2 1 1 3 2 1	:::״:.״
Srlma1 7״ 2 1 24 28 1 2 1 3 2 6 7 4 1 2 1־
Gastritis ------ 3	1	1 1	1	3 É	?
Hepatitis ............................... 1	2	2	*2
Hydrops......... 1	J	3	1 ״	ã
Hydrocele....... 1	ļ 1	3	2 1	3
Hydrothorax..... ״ 1 9 ״ ן
Hysteria........ 1	33	į
Hydrocephalus.	2	2	¿	¿
Icterus......... 1	2	, q
Irritatio Spinalis.... 11	ļ	!	2	1 ð
b"4 66 2 2 1 6 55 70 2 5 7 6 3 12 6 3 2 3 8 5 8 ::״״.״״
Keloides....................... 1	2	2	2
EUùTľºDS...... 11	2 3	11	j 2	12	4 3 4	Ц 1
, 2 4 2 1	3	2 Ş 4 4 4 S 3	40 3״	! β 3J 3
Metritis...... ť	1 1 .	9	0	2
Nephritis..... - ļ 5 1 4 5 1 ן
oļÄiľ:.':6 27 1 1 2 23 33 2 2 1 2 3 2 3 1 ’ 3 6 4 5 :־.:״
Orchitis...... 1	2 * נ i	2 I	2
Osteo Sarcoma-	*	!	~	ן
Paralysis...... 1	*	'	1	1	-	1
Parasites..................... .	1Ĵ	11
Pertussis.......У	4	·	4	<	-
(Table continued.)
ADMISSIONS.	DISCHARGES.
......ĩŗpfīfΠffΠi
Įļ¿ţ ((■· י i ··«·■·	(	I ■ ו *	·	“ Ç·Ķ
1Peritonitis................. 1	1	!	2 1	12
Phlebitis.................... 2	21	12
Phthisis....... 4 2 6 2 6	3	2	3 1	3	4	35 3 15 2 12 32 3
Pleuritis...... ,12	1	112	11	10 61	1191
Pneumonia ..... 1122	175	161
Polypus........ I 1	11	1
Pregnancies.... 112	2 1! 1 2 2 12 9	93
Rheumatism..... 21125 2 2 2 217 33 3236׳ δl 2 25 8
Scrofula....... 2	2	1	,'4	1	10	4 3	73
Sprains................ 1	2	3(	66	6
Stomatitis............  1	1	22	2
Strictures............  1	2	33	3
Syphilis....... 10 12 8 5 9 12	8	5 8 6 6 10 3 102 87 5 2 5	1 100 ~
Tumors........j	1	1	1	32	21
Tetanus...................... 1	1	2	22
Ulcers......... 10 4 6 2 61 2	3	2 3 6 1 4 4	53 43 7	1	51	2
Ulcers of the Uterus.	1	12	1	11
Ustio.............................. 1	1	4	1
Wounds ........ 3 2 3 1 2 1 1 1 3 3 1 21 17 2	19 2
Total..... 102 66 79 70 97 143 160110 72 81 71 79 57 1187 757 59 33 28 13511112 75
				

